# Poly[(μ_4_-biphenyl-3,3′-dicarboxyl­ato)bis[μ_2_-1,1′-(butane-1,4-di­yl)diimidazole](μ_2_-oxalato)dimanganese(II)]

**DOI:** 10.1107/S1600536810035269

**Published:** 2010-09-04

**Authors:** Bao-Yong Zhu

**Affiliations:** a Department of Chemistry, Dezhou University, Dezhou Shandong 253023, People’s Republic of China

## Abstract

In the title coordination compound, [Mn_2_(C_14_H_8_O_4_)(C_2_O_4_)(C_10_H_14_N_4_)_2_]_*n*_, the biphenyl-3,3′-dicarboxyl­ate and oxalate anions, both situated on inversion centres, function in a bridging mode, linking the dinuclear Mn^II^ atoms into wave-like layers. Each 1,1′-(1,4-butane-1,4-di­yl)diimidazole ligand coordinates to two Mn^II^ atoms located in adjacent layers *via* Mn—N coordination bonds, giving a three-dimensional network. As the methyl­ene groups can bend freely relative to each other due to the C atoms  connected *via* single bonds, the 1,1′-(butane-1,4-di­yl)diimidazole ligand forms an S-shaped conformation, which makes the void in the three-dimensional network distorted.

## Related literature

For the synthesis of the ligand, see: Yang *et al.* (2005 ▶). For the structures of related complexes, see: Wang *et al.* (2005 ▶). For related structures, see: Zhang *et al.* (2008 ▶); Zhou *et al.* (2009 ▶). 
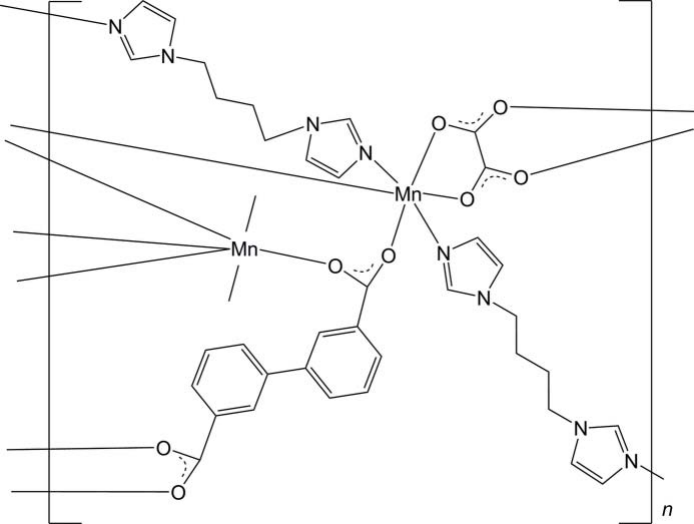


## Experimental

### 

#### Crystal data

[Mn_2_(C_14_H_8_O_4_)(C_2_O_4_)(C_10_H_14_N_4_)_2_]
*M* *_r_* = 818.60
Triclinic, 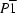

*a* = 9.532 (8) Å
*b* = 9.881 (8) Å
*c* = 11.051 (9) Å
α = 104.397 (2)°
β = 99.707 (2)°
γ = 114.265 (5)°
*V* = 874.8 (12) Å^3^
*Z* = 1
Mo *K*α radiation
μ = 0.79 mm^−1^
*T* = 296 K
0.13 × 0.11 × 0.10 mm

#### Data collection

Bruker APEXII CCD area-detector diffractometer
Absorption correction: multi-scan (*SADABS*; Bruker, 2001 ▶) *T*_min_ = 0.902, *T*_max_ = 0.923
4577 measured reflections
3063 independent reflections
2495 reflections with *I* > 2σ(*I*)
*R*_int_ = 0.020

#### Refinement

*R*[*F* ^2^ > 2σ(*F* ^2^)] = 0.039
*wR*(*F* ^2^) = 0.091
*S* = 1.01
3063 reflections
244 parameters
H-atom parameters constrained
Δρ_max_ = 0.71 e Å^−3^
Δρ_min_ = −0.36 e Å^−3^

### 

Data collection: *APEX2* (Bruker, 2007 ▶); cell refinement: *SAINT* (Bruker, 2007 ▶); data reduction: *SAINT* ; program(s) used to solve structure: *SIR97* (Altomare *et al.*, 1999 ▶); program(s) used to refine structure: *SHELXL97* (Sheldrick, 2008 ▶); molecular graphics: *SHELXTL* (Sheldrick, 2008 ▶); software used to prepare material for publication: *WinGX* (Farrugia, 1999 ▶).

## Supplementary Material

Crystal structure: contains datablocks global, I. DOI: 10.1107/S1600536810035269/hg2706sup1.cif

Structure factors: contains datablocks I. DOI: 10.1107/S1600536810035269/hg2706Isup2.hkl

Additional supplementary materials:  crystallographic information; 3D view; checkCIF report

## Figures and Tables

**Fig. 1.**
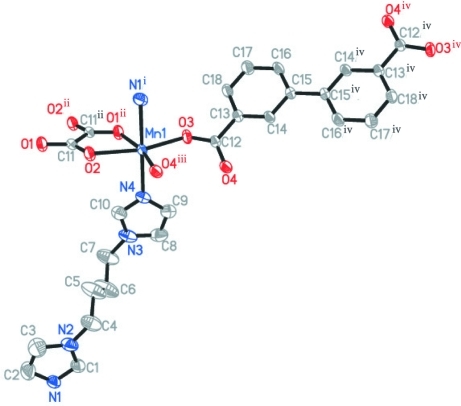
View of the MnII coordination environment of (I) with the atom-labelling scheme. Displacement ellipsoids are drawn at the 50% probability level. H atoms are represented by circles of arbitrary size. Symmetry code: (i) x, -1 + y, -1 + z; (ii) 2-x, 1-y, -z; (iii) 1-x, -y, -z; (iv) -x, -1 - y, -1-z.

**Fig. 2.**
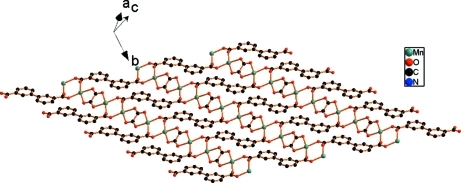
Perspective view of the two-dimensional layer of (I).

**Fig. 3.**
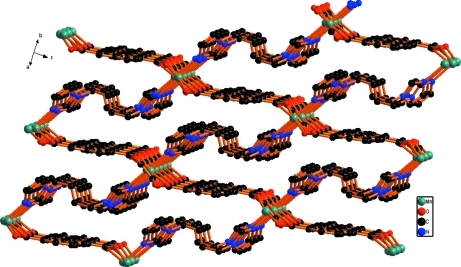
View of a three- dimensional framework of (I).

## supplementary crystallographic information

### Comment

Single crystal X-ray crystallographic analysis reveals that (I) crystallizes in triclinic system, space group P-1. It consists of dinuclear units of Mn^II^ atoms and the separation between the dinuclear Mn^II^ atoms is 4.823 (2) Å. The coordination environment around each Mn^II^ atom is shown in Figure 1 with the atom numbering scheme. In the asymmetric unit, there is one Mn^II^ atom, one 1,1'-(butane-1,4-diyl)diimidazole (bbi) ligand, half a 3, 3'-biphenyldicarboxylate (3, 3'-bpda) ligand and half an oxalate (ox) anion. The Mn^II^ atom is six-coordinated with distorted octahedral coordination geometry by two nitrogen (N1^i^, N4) atoms of two distinct bbi ligands with Mn - N bond lengths of 2.257 (5) and 2.261 (6) Å and four oxygen atoms (O1^ii^, O2, O3, O4^iii^) from two distinct 3, 3'-bpda ligands and one ox ligand with Mn-O bond lengths in the range of 2.234 (4)-2.155 (4) Å, and the coordination angles around Mn1 are in the range of 85.7 (2) to 178.2 (2) ° (Table 1). The ox anion coordinates to two Mn^II^ centers *via* a chelating bis-bidentate coordination mode and serves as a bridging ligand. 3, 3'-bpda ligand adopts a *trans* conformation and acts as a bis-bidentate bridging ligand, the carboxylate groups are slightly twisted with respect to correspondingly linking phenyl rings with the dihedral angles 6.5 and 9.5°.  The dihedral angle between two phenyl rings about the central bond is 0 °, which suggests the two phenyl rings are coplanar, showing a perfect *trans* conformation, which is different from that observed in metal-organic complexes reported previously (Wang *et al.*, 2005). The Mn^II^ atoms are linked by 3, 3'-bpda and ox ligands into wave-like two dimensional layers (Fig. 2). Each bbi ligand coordinates to two Mn^II^ centers located in adjacent layers *via* Mn-N coordination bonds to give rise to a three-dimensional network (Fig. 3).

It is noteworthy that the flexibility of the bbi ligand has a great influence to the framework. In structure of (I), because the methylene can bend freely to each other, the bbi ligand form a "S" shape conformation, which makes the void in the three-dimensional network distorted. This may reduce the surface energy of the framework and result in the uninterpenetrating three-dimensional framework of (I), unlike previously reported relate structures (Zhang *et al.*, 2008; Zhou *et al.*, 2009), which are composed of equivalent mutually interpenetrating networks. The distance between two Mn^II^ centers linked by bbi ligand is 12.87 Å, shorter than previously reported relevant structure (Zhang *et al.*, 2008).

### Experimental

The ligand bbi was synthesized according to the literature (Yang *et al.*, 2005). For the synthesis of (I), a mixture of 3, 3'-bpda (0.024 g, 0.1 mmol), bbi (0.021 g, 0.1 mmol), MnCl_2_4H_2_O (0.020 g, 0.1 mmol), and NaOH (0.008 g, 0.2 mmol) in H_2_O (7.0 ml) was placed in a 16 ml Teflon-lined stainless steel vessel and heated to 180 °C for 72 h to give rise to colorless block crystals of (I), which were collected by filtration. The colorless crystals obtained were washed with water and dried in air. Yield: 0.046 g (56% based on 3, 3'-bpda). IR (KBr pellet, cm^-1^): 3422(w), 3123(w), 1642(*s*), 1604(*s*), 1563(*s*), 1517(*m*), 1469(w), 1447(w), 1380(*s*), 1310(*s*), 1278(*m*), 1233(*s*), 1107(*s*), 1094(*s*), 936(*s*), 826(*s*), 782(*s*), 682(*m*), 657(*s*), 497(*m*).

### Refinement

All H atoms were added according to theoretical models, assigned isotropic displacement parameters and allowed to ride on their respective parent atoms[C—H=0.93–0.97%A and *U*_iso_=1.2*U*_eq_].

### Crystal data

**Table:**

[Mn_2_(C_14_H_8_O_4_)(C_2_O_4_)(C_10_H_14_N_4_)_2_]	*Z* = 1
*M**_r_* = 818.60	*F*(000) = 422
Triclinic, *P*1	*D*_x_ = 1.554 Mg m^−^^3^
Hall symbol: -P 1	Mo *K*α radiation, λ = 0.71073 Å
*a* = 9.532 (8) Å	Cell parameters from 1712 reflections
*b* = 9.881 (8) Å	θ = 2.5–27.1°
*c* = 11.051 (9) Å	µ = 0.79 mm^−^^1^
α = 104.397 (2)°	*T* = 296 K
β = 99.707 (2)°	Block, colorless
γ = 114.265 (5)°	0.13 × 0.11 × 0.10 mm
*V* = 874.8 (12) Å^3^	

### Data collection

**Table:**

Bruker APEXII CCD area-detector diffractometer	3063 independent reflections
Radiation source: fine-focus sealed tube	2495 reflections with *I* > 2σ(*I*)
graphite	*R*_int_ = 0.020
φ and ω scans	θ_max_ = 25.0°, θ_min_ = 2.0°
Absorption correction: multi-scan (*SADABS*; Bruker, 2001)	*h* = −9→11
*T*_min_ = 0.902, *T*_max_ = 0.923	*k* = −11→11
4577 measured reflections	*l* = −13→12

### Refinement

**Table:**

Refinement on *F*^2^	Primary atom site location: structure-invariant direct methods
Least-squares matrix: full	Secondary atom site location: difference Fourier map
*R*[*F*^2^ > 2σ(*F*^2^)] = 0.039	Hydrogen site location: inferred from neighbouring sites
*wR*(*F*^2^) = 0.091	H-atom parameters constrained
*S* = 1.01	*w* = 1/[σ^2^(*F*_o_^2^) + (0.0337*P*)^2^ + 0.7805*P*] where *P* = (*F*_o_^2^ + 2*F*_c_^2^)/3
3063 reflections	(Δ/σ)_max_ < 0.001
244 parameters	Δρ_max_ = 0.71 e Å^−^^3^
0 restraints	Δρ_min_ = −0.35 e Å^−^^3^

### Special details

**Table:**

Geometry. All e.s.d.'s (except the e.s.d. in the dihedral angle between two l.s. planes) are estimated using the full covariance matrix. The cell e.s.d.'s are taken into account individually in the estimation of e.s.d.'s in distances, angles and torsion angles; correlations between e.s.d.'s in cell parameters are only used when they are defined by crystal symmetry. An approximate (isotropic) treatment of cell e.s.d.'s is used for estimating e.s.d.'s involving l.s. planes.
Refinement. Refinement of *F*^2^ against ALL reflections. The weighted *R*-factor *wR* and goodness of fit *S* are based on *F*^2^, conventional *R*-factors *R* are based on *F*, with *F* set to zero for negative *F*^2^. The threshold expression of *F*^2^ > σ(*F*^2^) is used only for calculating *R*-factors(gt) *etc*. and is not relevant to the choice of reflections for refinement. *R*-factors based on *F*^2^ are statistically about twice as large as those based on *F*, and *R*- factors based on ALL data will be even larger.

### Fractional atomic coordinates and isotropic or equivalent isotropic displacement parameters (Å^2^)

**Table:**

	*x*	*y*	*z*	*U*_iso_*/*U*_eq_	
C1	0.7299 (4)	0.9282 (4)	0.7716 (3)	0.0427 (8)	
H1	0.6181	0.8815	0.7375	0.051*	
C2	0.9736 (4)	1.0911 (5)	0.8827 (4)	0.0633 (11)	
H2	1.0655	1.1814	0.9418	0.076*	
C3	0.9734 (5)	0.9615 (5)	0.8048 (4)	0.0713 (12)	
H3	1.0628	0.9464	0.8006	0.086*	
C4	0.7560 (5)	0.6974 (4)	0.6402 (3)	0.0564 (10)	
H4A	0.6452	0.6333	0.6363	0.068*	
H4B	0.8193	0.6507	0.6722	0.068*	
C5	0.7612 (6)	0.6915 (5)	0.5041 (4)	0.0768 (13)	
H5A	0.8727	0.7552	0.5093	0.092*	
H5B	0.7285	0.5827	0.4517	0.092*	
C6	0.6637 (6)	0.7444 (5)	0.4343 (4)	0.0816 (14)	
H6A	0.6957	0.8530	0.4863	0.098*	
H6B	0.5517	0.6801	0.4278	0.098*	
C7	0.6733 (6)	0.7376 (4)	0.2962 (4)	0.0642 (11)	
H7A	0.6072	0.7796	0.2600	0.077*	
H7B	0.7841	0.8044	0.3016	0.077*	
C8	0.4624 (4)	0.4587 (4)	0.1455 (3)	0.0505 (9)	
H8	0.3691	0.4661	0.1502	0.061*	
C9	0.4710 (4)	0.3296 (4)	0.0757 (3)	0.0444 (8)	
H9	0.3825	0.2319	0.0237	0.053*	
C10	0.7130 (4)	0.5128 (4)	0.1726 (3)	0.0423 (8)	
H10	0.8254	0.5686	0.2016	0.051*	
C11	1.0488 (3)	0.4969 (3)	0.0624 (2)	0.0253 (6)	
C12	0.3716 (3)	−0.0489 (3)	−0.1928 (2)	0.0240 (6)	
C13	0.2848 (3)	−0.1643 (3)	−0.3312 (2)	0.0262 (6)	
C14	0.1514 (3)	−0.3102 (3)	−0.3588 (2)	0.0257 (6)	
H14	0.1146	−0.3347	−0.2904	0.031*	
C15	0.0709 (3)	−0.4210 (3)	−0.4862 (3)	0.0261 (6)	
C16	0.1284 (3)	−0.3790 (3)	−0.5861 (3)	0.0344 (7)	
H16	0.0772	−0.4505	−0.6720	0.041*	
C17	0.2597 (4)	−0.2338 (3)	−0.5611 (3)	0.0408 (8)	
H17	0.2949	−0.2082	−0.6298	0.049*	
C18	0.3386 (4)	−0.1266 (3)	−0.4339 (3)	0.0352 (7)	
H18	0.4276	−0.0293	−0.4169	0.042*	
Mn1	0.72380 (5)	0.21542 (5)	−0.02078 (4)	0.02532 (14)	
N1	0.8196 (3)	1.0703 (3)	0.8619 (2)	0.0365 (6)	
N2	0.8173 (3)	0.8581 (3)	0.7341 (2)	0.0443 (7)	
N3	0.6182 (4)	0.5751 (3)	0.2074 (2)	0.0451 (7)	
N4	0.6292 (3)	0.3645 (3)	0.0931 (2)	0.0365 (6)	
O1	1.1919 (2)	0.6036 (2)	0.11609 (18)	0.0324 (5)	
O2	0.9760 (2)	0.3849 (2)	0.09977 (17)	0.0318 (5)	
O3	0.5021 (2)	0.0710 (2)	−0.17499 (18)	0.0326 (5)	
O4	0.3098 (2)	−0.0764 (2)	−0.10413 (17)	0.0310 (4)	

### Atomic displacement parameters (Å^2^)

**Table:**

	*U*^11^	*U*^22^	*U*^33^	*U*^12^	*U*^13^	*U*^23^
C1	0.0386 (18)	0.0441 (18)	0.0401 (18)	0.0215 (16)	0.0121 (14)	0.0027 (15)
C2	0.039 (2)	0.064 (2)	0.066 (3)	0.0244 (19)	0.0084 (18)	−0.004 (2)
C3	0.053 (2)	0.078 (3)	0.079 (3)	0.043 (2)	0.017 (2)	0.001 (2)
C4	0.082 (3)	0.046 (2)	0.047 (2)	0.041 (2)	0.0196 (19)	0.0089 (16)
C5	0.126 (4)	0.066 (3)	0.044 (2)	0.062 (3)	0.018 (2)	0.0044 (19)
C6	0.136 (4)	0.067 (3)	0.052 (3)	0.071 (3)	0.018 (3)	0.004 (2)
C7	0.111 (3)	0.048 (2)	0.047 (2)	0.052 (2)	0.025 (2)	0.0127 (17)
C8	0.054 (2)	0.071 (2)	0.0406 (19)	0.042 (2)	0.0193 (17)	0.0181 (18)
C9	0.046 (2)	0.0482 (19)	0.0398 (18)	0.0234 (17)	0.0171 (15)	0.0127 (15)
C10	0.0470 (19)	0.0421 (18)	0.0361 (17)	0.0230 (16)	0.0115 (14)	0.0084 (14)
C11	0.0246 (14)	0.0209 (13)	0.0227 (14)	0.0074 (12)	0.0068 (11)	0.0017 (11)
C12	0.0223 (14)	0.0221 (13)	0.0213 (14)	0.0079 (12)	0.0028 (11)	0.0048 (11)
C13	0.0234 (14)	0.0239 (13)	0.0238 (14)	0.0082 (11)	0.0036 (11)	0.0044 (11)
C14	0.0272 (14)	0.0229 (13)	0.0194 (13)	0.0084 (12)	0.0040 (11)	0.0037 (10)
C15	0.0232 (14)	0.0218 (13)	0.0237 (14)	0.0064 (12)	0.0034 (11)	0.0032 (11)
C16	0.0364 (16)	0.0276 (15)	0.0199 (14)	0.0046 (13)	0.0030 (12)	0.0004 (12)
C17	0.0437 (18)	0.0343 (16)	0.0259 (16)	0.0031 (14)	0.0120 (13)	0.0076 (13)
C18	0.0347 (16)	0.0252 (15)	0.0270 (15)	0.0007 (13)	0.0084 (12)	0.0041 (12)
Mn1	0.0227 (2)	0.0210 (2)	0.0202 (2)	0.00355 (17)	0.00432 (16)	0.00186 (16)
N1	0.0367 (14)	0.0374 (14)	0.0309 (13)	0.0189 (12)	0.0091 (11)	0.0032 (11)
N2	0.0532 (17)	0.0477 (16)	0.0342 (15)	0.0306 (14)	0.0137 (13)	0.0059 (12)
N3	0.069 (2)	0.0442 (16)	0.0321 (14)	0.0374 (16)	0.0166 (14)	0.0108 (12)
N4	0.0448 (15)	0.0349 (14)	0.0277 (13)	0.0205 (12)	0.0116 (11)	0.0045 (11)
O1	0.0249 (10)	0.0301 (10)	0.0270 (10)	0.0030 (9)	0.0014 (8)	0.0082 (8)
O2	0.0292 (11)	0.0268 (10)	0.0242 (10)	0.0028 (9)	0.0028 (8)	0.0073 (8)
O3	0.0240 (10)	0.0249 (10)	0.0291 (10)	−0.0001 (9)	0.0022 (8)	0.0029 (8)
O4	0.0338 (11)	0.0268 (10)	0.0217 (10)	0.0076 (9)	0.0068 (8)	0.0047 (8)

### Geometric parameters (Å, °)

**Table:**

C1—N1	1.314 (4)	C10—H10	0.9300
C1—N2	1.339 (4)	C11—O1	1.248 (3)
C1—H1	0.9300	C11—O2	1.252 (3)
C2—C3	1.353 (5)	C11—C11^i^	1.559 (5)
C2—N1	1.366 (4)	C12—O4	1.256 (3)
C2—H2	0.9300	C12—O3	1.257 (3)
C3—N2	1.352 (5)	C12—C13	1.502 (3)
C3—H3	0.9300	C13—C18	1.392 (4)
C4—N2	1.467 (4)	C13—C14	1.390 (4)
C4—C5	1.501 (5)	C14—C15	1.396 (4)
C4—H4A	0.9700	C14—H14	0.9300
C4—H4B	0.9700	C15—C16	1.392 (4)
C5—C6	1.446 (6)	C15—C15^ii^	1.500 (5)
C5—H5A	0.9700	C16—C17	1.382 (4)
C5—H5B	0.9700	C16—H16	0.9300
C6—C7	1.532 (5)	C17—C18	1.383 (4)
C6—H6A	0.9700	C17—H17	0.9300
C6—H6B	0.9700	C18—H18	0.9300
C7—N3	1.472 (4)	Mn1—O3	2.1222 (19)
C7—H7A	0.9700	Mn1—O4^iii^	2.151 (2)
C7—H7B	0.9700	Mn1—O2	2.205 (2)
C8—C9	1.360 (5)	Mn1—O1^i^	2.235 (2)
C8—N3	1.364 (4)	Mn1—N4	2.263 (2)
C8—H8	0.9300	Mn1—N1^iv^	2.263 (2)
C9—N4	1.368 (4)	N1—Mn1^v^	2.263 (2)
C9—H9	0.9300	O1—Mn1^i^	2.235 (2)
C10—N4	1.311 (4)	O4—Mn1^iii^	2.151 (2)
C10—N3	1.341 (4)		
			
N1—C1—N2	112.6 (3)	C18—C13—C12	120.0 (2)
N1—C1—H1	123.7	C14—C13—C12	120.9 (2)
N2—C1—H1	123.7	C13—C14—C15	122.0 (2)
C3—C2—N1	110.2 (3)	C13—C14—H14	119.0
C3—C2—H2	124.9	C15—C14—H14	119.0
N1—C2—H2	124.9	C16—C15—C14	117.3 (2)
C2—C3—N2	106.4 (3)	C16—C15—C15^ii^	121.5 (3)
C2—C3—H3	126.8	C14—C15—C15^ii^	121.2 (3)
N2—C3—H3	126.8	C17—C16—C15	121.7 (2)
N2—C4—C5	114.0 (3)	C17—C16—H16	119.1
N2—C4—H4A	108.8	C15—C16—H16	119.1
C5—C4—H4A	108.8	C18—C17—C16	120.0 (3)
N2—C4—H4B	108.8	C18—C17—H17	120.0
C5—C4—H4B	108.8	C16—C17—H17	120.0
H4A—C4—H4B	107.7	C17—C18—C13	119.9 (3)
C6—C5—C4	117.6 (4)	C17—C18—H18	120.0
C6—C5—H5A	107.9	C13—C18—H18	120.0
C4—C5—H5A	107.9	O3—Mn1—O4^iii^	99.95 (8)
C6—C5—H5B	107.9	O3—Mn1—O2	165.44 (7)
C4—C5—H5B	107.9	O4^iii^—Mn1—O2	93.16 (7)
H5A—C5—H5B	107.2	O3—Mn1—O1^i^	92.32 (7)
C5—C6—C7	116.0 (4)	O4^iii^—Mn1—O1^i^	167.71 (7)
C5—C6—H6A	108.3	O2—Mn1—O1^i^	74.74 (7)
C7—C6—H6A	108.3	O3—Mn1—N4	93.96 (9)
C5—C6—H6B	108.3	O4^iii^—Mn1—N4	91.30 (9)
C7—C6—H6B	108.3	O2—Mn1—N4	92.05 (9)
H6A—C6—H6B	107.4	O1^i^—Mn1—N4	87.11 (9)
N3—C7—C6	112.3 (3)	O3—Mn1—N1^iv^	85.82 (9)
N3—C7—H7A	109.1	O4^iii^—Mn1—N1^iv^	90.21 (9)
C6—C7—H7A	109.1	O2—Mn1—N1^iv^	87.83 (9)
N3—C7—H7B	109.1	O1^i^—Mn1—N1^iv^	91.42 (9)
C6—C7—H7B	109.1	N4—Mn1—N1^iv^	178.50 (9)
H7A—C7—H7B	107.9	C1—N1—C2	104.3 (3)
C9—C8—N3	106.0 (3)	C1—N1—Mn1^v^	125.1 (2)
C9—C8—H8	127.0	C2—N1—Mn1^v^	129.6 (2)
N3—C8—H8	127.0	C1—N2—C3	106.6 (3)
C8—C9—N4	110.0 (3)	C1—N2—C4	127.0 (3)
C8—C9—H9	125.0	C3—N2—C4	126.4 (3)
N4—C9—H9	125.0	C10—N3—C8	106.7 (3)
N4—C10—N3	112.3 (3)	C10—N3—C7	126.2 (3)
N4—C10—H10	123.9	C8—N3—C7	127.0 (3)
N3—C10—H10	123.9	C10—N4—C9	105.0 (3)
O1—C11—O2	126.0 (2)	C10—N4—Mn1	127.0 (2)
O1—C11—C11^i^	117.2 (3)	C9—N4—Mn1	127.0 (2)
O2—C11—C11^i^	116.8 (3)	C11—O1—Mn1^i^	115.06 (17)
O4—C12—O3	124.7 (2)	C11—O2—Mn1	116.18 (16)
O4—C12—C13	118.9 (2)	C12—O3—Mn1	135.35 (17)
O3—C12—C13	116.5 (2)	C12—O4—Mn1^iii^	143.26 (18)
C18—C13—C14	119.1 (2)		
			
N1—C2—C3—N2	0.1 (5)	C6—C7—N3—C10	−101.2 (4)
N2—C4—C5—C6	−63.2 (5)	C6—C7—N3—C8	78.4 (5)
C4—C5—C6—C7	179.6 (4)	N3—C10—N4—C9	0.1 (4)
C5—C6—C7—N3	61.2 (6)	N3—C10—N4—Mn1	169.41 (19)
N3—C8—C9—N4	−0.2 (4)	C8—C9—N4—C10	0.0 (4)
O4—C12—C13—C18	171.3 (3)	C8—C9—N4—Mn1	−169.3 (2)
O3—C12—C13—C18	−7.9 (4)	O3—Mn1—N4—C10	−151.4 (3)
O4—C12—C13—C14	−9.4 (4)	O4^iii^—Mn1—N4—C10	108.5 (3)
O3—C12—C13—C14	171.5 (2)	O2—Mn1—N4—C10	15.3 (3)
C18—C13—C14—C15	0.9 (4)	O1^i^—Mn1—N4—C10	−59.3 (3)
C12—C13—C14—C15	−178.5 (2)	O3—Mn1—N4—C9	15.6 (3)
C13—C14—C15—C16	−0.8 (4)	O4^iii^—Mn1—N4—C9	−84.5 (2)
C13—C14—C15—C15^ii^	178.8 (3)	O2—Mn1—N4—C9	−177.7 (2)
C14—C15—C16—C17	0.0 (4)	O1^i^—Mn1—N4—C9	107.7 (2)
C15^ii^—C15—C16—C17	−179.6 (3)	O2—C11—O1—Mn1^i^	−179.1 (2)
C15—C16—C17—C18	0.7 (5)	C11^i^—C11—O1—Mn1^i^	0.8 (4)
C16—C17—C18—C13	−0.6 (5)	O1—C11—O2—Mn1	−179.2 (2)
C14—C13—C18—C17	−0.2 (4)	C11^i^—C11—O2—Mn1	0.9 (4)
C12—C13—C18—C17	179.2 (3)	O3—Mn1—O2—C11	26.9 (4)
N2—C1—N1—C2	0.0 (4)	O4^iii^—Mn1—O2—C11	−178.85 (18)
N2—C1—N1—Mn1^v^	−169.0 (2)	O1^i^—Mn1—O2—C11	−1.00 (17)
C3—C2—N1—C1	−0.1 (5)	N4—Mn1—O2—C11	−87.44 (19)
C3—C2—N1—Mn1^v^	168.3 (3)	N1^iv^—Mn1—O2—C11	91.06 (19)
N1—C1—N2—C3	0.0 (4)	O4—C12—O3—Mn1	33.5 (4)
N1—C1—N2—C4	177.0 (3)	C13—C12—O3—Mn1	−147.35 (19)
C2—C3—N2—C1	−0.1 (5)	O4^iii^—Mn1—O3—C12	9.5 (3)
C2—C3—N2—C4	−177.1 (3)	O2—Mn1—O3—C12	163.4 (3)
C5—C4—N2—C1	97.7 (4)	O1^i^—Mn1—O3—C12	−169.7 (2)
C5—C4—N2—C3	−85.9 (5)	N4—Mn1—O3—C12	−82.5 (3)
N4—C10—N3—C8	−0.2 (4)	N1^iv^—Mn1—O3—C12	99.0 (3)
N4—C10—N3—C7	179.4 (3)	O3—C12—O4—Mn1^iii^	−102.2 (3)
C9—C8—N3—C10	0.2 (4)	C13—C12—O4—Mn1^iii^	78.7 (3)
C9—C8—N3—C7	−179.4 (3)		

Symmetry codes: (i) −*x*+2, −*y*+1, −*z*; (ii) −*x*, −*y*−1, −*z*−1; (iii) −*x*+1, −*y*, −*z*; (iv) *x*, *y*−1, *z*−1; (v) *x*, *y*+1, *z*+1.
